# Retraction: Mesenchymal Stromal Cells for Tissue-Engineered Tissue and Organ Replacements

**DOI:** 10.3389/ti.2024.12843

**Published:** 2024-03-05

**Authors:** Transplant International Editorial Office

Transplant International published in 2012 a review paper [1], in which another article from the same authors was cited [2]. This article had been published in the Lancet in 2008 and was very recently retracted for demonstrated falsification [3]. The falsification had been confirmed in a decision by the Swedish National Board for Assessment of Research Misconduct [4].

Briefly, the paper retracted by the Lancet described the allegedly successful transplantation of a tissue-engineered tracheal segment, reporting that “the graft immediately provided the recipient with a functional airway, improved her quality of life, and had a normal appearance and mechanical properties at 4 months” [2]. The conclusions of the investigation by the Swedish National Board for Assessment of Research Misconduct found that this statement constituted falsification [4].

The review article [1] was accepted and published in Transplant International based on its perceived quality and the supposed merits of the authors’ work. In this review, the authors stated that “(tissue engineering) has already provided functional tissue and organ human replacement” (sic), citing to support this point the retracted Lancet article [2] that they knew contained falsified data.

We are therefore retracting this review article.

Thierry Berney.

Transplant International editor-in-chief.
